# The status of adolescent health during the COVID-19 pandemic

**DOI:** 10.25122/jml-2021-0287

**Published:** 2022-05

**Authors:** Seideh Hanieh Alamolhoda, Elham Zare, Mliheh Nasiri

**Affiliations:** 1. Department of Midwifery and Reproductive Health, Midwifery and Reproductive Health Research Center, School of Nursing and Midwifery, Shahid Beheshti University of Medical Sciences, Tehran, Iran; 2. Department of Biostatistics, School of Nursing and Midwifery, Shahid Beheshti University of Medical Sciences, Tehran, Iran

**Keywords:** COVID-19, adolescent health, anxiety

## Abstract

Coronavirus is a life-threatening disease with many devastating psychological, emotional, social, and sexual implications, especially for vulnerable people. This cross-sectional study aimed to assess adolescent health and anxiety during the COVID-19 pandemic. 1300 male adolescents filled out the Male Adolescent Health Need Assessment Scale (MAHNAS) and corona-related anxiety scale (CRAS) from January to May 2021. Data analysis was done using SPSS version 22. The mean age of adolescents was 15.5±2.25, and most of them (65%) were studying in high school. The mean scores for physical health were 78±7.55, psychological health 48.8±5.55, social health 48.21±8.61, sexual health 50.35±9.05, and anxiety scores were severe (46.76) in this study. There was a significant negative relationship between anxiety and health needs. The COVID-19 pandemic greatly affected the health needs of adolescents. Effective approaches and policies in this crisis, especially for adolescents, can alleviate the anxious reactions and be a way to meet their health needs appropriately.

## INTRODUCTION

The World Health Organization (WHO) announced a health status crisis of international concern in 2020. SARS-CoV-2 epidemic is becoming a global problem [1, 2]. Inhalation or contact with infected droplets is the main way of transmission. Fever, fatigue, sore throat, cough, breathlessness, and malaise are the most common symptoms. The case fatality rate is about 2 to 3%, with all ages vulnerable [3, 4].

There is no special treatment so far. The logical way to cope with SARS-CoV-2 is by controlling the source of infection, guarding vulnerable people, and cutting off the transmission. People should take measures to protect themselves, such as home quarantine, social contacts limitation, and use a mask in public places [5]. In addition, the authorities should recommend people to stay home, discourage mass gatherings, delay or cancel public events, and public institutions must be closed [3].

According to the WHO declaration, this universal situation may negatively impact the well-being of individuals, which in turn impacts individuals' performance [6]. Previous data on mass happenings, like natural disasters, shows that troublesome large-scale events are strongly associated with negative effects on health status and are followed by other behavioral and psychological chaos [7].

One of the most vulnerable age groups who suffered greatly from this crisis is adolescents. The period between childhood and adulthood, from ages 10 to 19, has been identified as adolescence. It is a unique phase of human development and a unique time for laying the foundations of good health [8].

However, the COVID-19 pandemic is putting the health and well-fare of all children and adolescents in danger. Injustice in this field is increasing every day, and there are no suitable health interventions for adolescents, adolescents being a vulnerable segment of society. Schools were closed in many countries at the height of lockdown, affecting 1.6 billion students [9]. Peer support groups and face-to-face services have been postponed, and cell phone or online support can be demanding for some young people. Becoming overweight among adolescents and children was getting worse, and acute stress disorder was more likely to develop in isolated or quarantined children during the pandemic [10]. The aim of this study was to assess the health needs of adolescents during the pandemic. Due to the lack of sufficient studies in this field, it is necessary to identify the health needs to deliver the requirements for proper planning and policy-making to promote adolescent health.

## MATERIAL AND METHODS

This study had a cross-sectional correlational design and recruited 1300 male adolescents (13–19 y/o) studying in high schools from January to May 2021. Inclusion criteria were not being married and studying in public schools. The exclusion criteria were declining to participate after completing the questionnaire. The sampling method was random multistage, and we used the table of random numbers with Tehran schools. The number of samples based on the proportion of adolescents living in each area was estimated until the total sample was completed. Online classes were also randomly selected in each school, and students were requested to fill out data collection tools online or offline according to their preferences. The demographic questionnaire and the Male Adolescent Health Need Assessment Scale (MAHNAS) were used. The MAHNAS consisted of 49 questions under the four dimensions of “sexual health”, “physical health”, “psychological health”, and “social health” with a 5-point Likert type scale (never=1, ever=5). The scores obtained ranged from 49 to 245, with higher scores indicating a better health condition and lower needs for health supervision. The psychometric properties of this scale were controlled using Cronbach's α coefficient of .79 and intraclass correlation index of .89 using a test-retest method [11]. The corona-related anxiety scale (CRAS) was also used, comprising 18 items with 4-point Likert answers (never=0, ever=3). The score of the corona-related anxiety severity was divided into three domains: mild (0–16), moderate (17–29), and severe (54–30). The Guttmann's λ2 value for the whole questionnaire was obtained as λ=0.922, Cronbach's alpha coefficient for psychological symptoms as α=0.879, physical symptoms as α=0.861, and the whole questionnaire as α=0.919 [12]. The collected data were analyzed using descriptive and t-test, one-way analysis of variance, and the Pearson correlation coefficient statistics via the SPSS Version 22.

## RESULTS

The mean age and standard deviation of 1300 male adolescents in this study were 15.5±2.25 years. Most of them were in high school (65%), had mothers with diplomas (41.2%), and fathers with bachelor's degrees (37.8%). Most mothers were employed (33%), and fathers were business owners (41.9%). According to the Kolmogorov Smirnov test, the data had normal distribution, and the large sample size allowed us to use parametric statistical tests. Table 1 shows the mean scores of MANHAS in each domain.

**Table 1:** The MANHAS scores are based on the score of 100 in each domain.

Domain	Mean (standard deviation)	Highest score	Lowest score
**Physical health need**	78 (±7.55)	96	58
**Psychological health need**	48.8 (±5.55)	68	30
**Social health need**	48.21 (±8.61)	70	30
**Sexual health need**	50.35 (±9.05)	60	41
**Total health need**	49.34 (±5.05)	61	38

Table 1 shows that physical and social health have higher scores than sexual and psychological health. In other words, sexual and psychological health domains need more attention to improve. Lower scores indicate higher health needs. The MANHAS instrument got an average score of 49.34. Table 2 demonstrates the number and percent of adolescents according to the severity of corona-related anxiety.

**Table 2:** Descriptive characteristic of CRAS in male adolescents.

Samples (n=1300)	Mild (0–16)	Moderate (17–29)	Severe (30–54)
**Adolescent**	105 (8.09%)	587 (45.15%)	608 (46.76)

Table 2 shows that most adolescents suffer from severe anxiety of coronavirus (46.76%), and just a few of them (8.09%) consider coronavirus a mild stressor in their life. Consequently, this novel virus represents a strong stressor for adolescents. Table 3 shows the significant correlation between MANHAS and CRAS scores in 1300 male adolescents.

**Table 3:** Correlation between MANHAS and CRAS scores.

CRAS score	Physical health need	Physiological health need	Social health need	Sexual health need	Total health need
**Pearson correlation (r)**	-.018	-0.49	-0.43	-0.19	-0.31
**P-value**	<0.001	<0.001	<0.001	<0.009	<0.001
**Number**	1300	1300	1300	1300	1300

Table 3 shows a significant negative correlation between all domains of MANHAS and anxiety of coronavirus. Higher anxiety is accompanied by a lower score and greater needs are apparent. The physiological and social needs had a stronger correlation with anxiety than physical and sexual health needs.

## DISCUSSION

This study aimed to evaluate the health needs of male adolescents during the COVID-19 pandemic. Most research on Covid-19 has been focused on adults and less on adolescents. This study reported a high prevalence of mental health problems in adolescents. Moreover, valid and reliable tools used to assess adolescents' health need status during the Covid-19 crisis can identify the real health needs of adolescents and inform policy-making. The large sample size and sampling of various parts of the city and including various health domain needs were other positive hallmarks of this study.

According to the results, the health needs of the physical dimension were favorable, and this may be since, during the Covid-19 crisis, individuals were more observant of their private and public health, such as washing hands, wearing masks, and forced distance. Proper nutritional status, especially to strengthen the immune system during the Covid-19, was another measure of good physical health status in this study. Furthermore, in a previous study in Iran, the state of physical health was also estimated to be in good condition [13].

One study in Ireland evaluated the physical activity of adolescents during the Covid-19 pandemic and declared that adolescents reported less physical activity (50%), no change (30%), or did more physical activity during lockdown (20%). Adolescents who did less physical activity were more likely to be overweight [14].

On the other hand, many social and psychological health needs were evident during the Covid-19 crisis among Iranian adolescents. In fact, most of the health needs in this area were reported. The Covid-19 crisis has increased these needs, and mental health services and interventions should be planned in public and private institutions to reduce the crisis in adolescents. The COVID-19 outburst triggered difficulties for child psychiatry and mental health services that must be directed, including public institutions covering interventions for major public health crises affecting youth. A greater challenge for this age group is dealing with lockdown and quarantine, which may push children into calamity and weaken families, especially when severe quarantine exists in the mentally or psychologically vulnerable [15]. Young people have to be separated from their loved ones during quarantine, afraid of their family and friends and need to cope with the Covid-19 crisis. These factors affect health, especially psychological health and well-being [16].

Many studies indicated that restrictions and limitations in previous pandemics and epidemics put pressure, stress, anxiety, emotional fatigue, post-traumatic stress disorder, and fear on the human being. Adolescents experience other severe stressors during a pandemic lockdown and quarantine, such as stress, infection, death, imprisonment, social contact limitation, helplessness, depression, anxiety, panic attacks, and even suicidality [17–19].

Several investigations in China declared that the rate of depression, anxiety, and psychological stress during COVID-19 was higher in children and adolescents [20–22]. Some researchers suggest various methods to alleviate these negative effects; for example, in Brazil, doing more activity and working out could alleviate mental health disorders in youth. Some factors significantly reduce mental distress related to Covid-19, such as physical exercise, multimedia, books, and physical activity [23, 24].

Social factors in the health of adolescents have always played an undeniable role. Adolescents' health generally refers to physical health and neglected social health. Epidemiological transmission changed the profile of adolescents' health needs. Lack of knowledge and attention to adolescents' health needs leads to high-risk situations, increased addiction rates in adolescents, academic failure, and psychosocial disorders. Adolescent mortality is not common; instead, the global burden of disease and disability is greater. Adolescent health crises need to be addressed and prevented on time [13]. According to a study previously performed in Iran to promote psychological and social health, community, family, schools, and mass media empowerment is essential for health authorities and good health policies. All these factors that affect vulnerable youth are more important in the Covid-19 crisis; therefore, more serious attention from policymakers is recommended in Iran [25, 26].

Another aspect of health needs is sexual health, which was not in a good or bad situation. According to the literature, sexual education has been less observed in the real context. Half of the adolescents' sexual problems are ignored in Iran [27, 28]. The current attention is also placed on boys' needs to formally gain more information and receive health education [27]. Covid-19 has made the situation worse than before. The lack of specific policies on adolescent sexual health education and restrictive rules for schools showed that sexual health issues were not prioritized in developing programs by government authorities in the Ministry of Health and Medical Education and the Department of Education [29].

Unfortunately, children are out of control of the health system from the age of eight and do not enter until middle age obligatory. Many researchers similarly believed that sexual health policies needed to be revised. Considering appropriate ideas, interests, strategies, and tactics to perform evidence-based health interventions in sexual and reproductive health is mandatory [30, 31].

In this study, coronavirus anxiety affected all aspects of teenage health. Obviously, fears concerning health are a typical response in the face of the Covid-19 pandemic. Children and young people are often exposed to death messages from the media and authorities and Covid-19 will probably be the first major global health threat to young people during the pandemic. This fear jeopardizes adolescents' and children's beliefs that the world is a safe place to live [32]. Therefore, providing an effective, empathic communication system with the direct participation of teenagers is important. Finally, psychological counseling services for the stress management of adolescents may help them to survive [33].

## CONCLUSION

In our study, all domains of adolescent health were affected by the Covid-19 except for physical health. Therefore, more attention and policies are needed to meet the needs of adolescents in Iran.
